# Dislocated Intraocular Lens Extraction and Iris-Claw Lens Implantation in Vitrectomized and Non-vitrectomized Eyes

**DOI:** 10.4274/tjo.galenos.2019.79735

**Published:** 2019-10-24

**Authors:** M. Giray Ersöz, Mümin Hocaoğlu, Işıl Bahar Sayman Muslubaş, Serra Arf, Murat Karaçorlu

**Affiliations:** 1 İstanbul Retina Institute, İstanbul, Turkey

**Keywords:** Aphakia, intraocular lens dislocation, iris-claw lens, secondary lens implantation, vitrectomy

## Abstract

**Objectives:**

To compare the outcomes and complications of dislocated intraocular lens (IOL) extraction and secondary iris-claw IOL (ICIOL) implantation in vitrectomized and non-vitrectomized eyes.

**Materials and Methods:**

This retrospective study included 19 vitrectomized eyes and 11 non-vitrectomized eyes that underwent dislocated IOL extraction and secondary anterior chamber ICIOL implantation between June 2014 and September 2017 and had at least one year of follow-up.

**Results:**

There were no significant differences between the groups in terms of demographic data, operative time, baseline anatomic and functional measurements, or postoperative changes in these measurements (all p>0.05). Postoperative best corrected visual acuity was significantly higher than preoperative values in both groups (both p<0.05). Complication rates did not differ between the groups (all p>0.05). In both groups, endothelial cell density was significantly lower at postoperative 1 year compared to preoperative measurements. There was no significant difference between groups regarding endothelial cell loss (p=0.49). One vitrectomized eye had corneal decompensation. Other complications included hyphema, transient increase of intraocular pressure, secondary glaucoma, pupillary irregularity, and dislocation of ICIOL. Mean operative time was 26.4±5.9 minutes.

**Conclusion:**

Dislocated IOL extraction and secondary anterior chamber ICIOL implantation is a safe treatment option in both vitrectomized and non-vitrectomized eyes.

## Introduction

In cataract surgery, implantation of the intraocular lens (IOL) in the capsule is the ideal position and provides excellent visual outcomes. With the introduction of multifocal and toric IOLs, cataract surgery has now become a form of refractive surgery and is performed at earlier ages. In patients who sustain capsular damage during cataract surgery but have adequate capsular support, monofocal IOLs can be placed in the sulcus in the posterior chamber. For cases with inadequate capsular support or dislocated intraocular or crystalline lens due to zonular damage, options include the use of an angle-supported anterior chamber IOL (ACIOL), a posterior chamber IOL fixated to the sclera or sutured to the iris, or iris-claw IOL (ICIOL).^1^

Although ACIOL implantation is an easy and rapid procedure, the risk of corneal decompensation and secondary glaucoma is higher than with other methods.^2^ Scleral fixation of a posterior chamber IOL is more similar to normal anatomic position of the lens. However, it is a longer and more difficult procedure. It also carries risks such as retinal detachment, choroidal hemorrhage, pigment dispersion, IOL decentration, and cystoid macular edema, as well as conjunctival erosion and endophthalmitis if transscleral suturing is used.^3,4^ Suturing posterior chamber IOLs to the iris is also not commonly performed due to factors such as its technical difficulty, long operative time, and high complication rates.^5^ ICIOL implantation, on the other hand, is easier, quicker, and associated with low intraoperative and postoperative complication rates.^6^ Although designed primarily for placement on the anterior surface of the iris, retropupillary placement is also possible.^7^

In addition to ocular trauma, pseudoexfoliation syndrome, high myopia, uveitis, and retinitis pigmentosa, a history of pars plana vitrectomy is also a risk factor for zonular dialysis.^8^ Because vitrectomized eyes lose the support provided by the vitreous, intraocular pressure (IOP) is difficult to maintain during surgery and the risk of suprachoroidal hemorrhage increases, especially in prolonged surgeries.^9^ In this study, we aimed to compare the outcomes and complications of dislocated IOL extraction with simultaneous ICIOL implantation in vitrectomized and non-vitrectomized eyes.

## Materials and Methods

This retrospective study included 19 vitrectomized eyes (group 1) and 11 non-vitrectomized eyes (group 2) that underwent IOL removal due to IOL dislocation and secondary ICIOL implantation to the anterior chamber and were followed up for at least 1 year at the Istanbul Retina Institute between June 2014 and September 2017. The study protocol was prepared in accordance with the Declaration of Helsinki and approved by the İstanbul Şişli Memorial Hospital Ethics Committee. Patient records were reviewed for the following data: medical history, systemic diseases, age, sex, previous ocular surgeries, surgical procedure, operative time, best corrected visual acuity (BCVA), spherical equivalent refractive error (SERE), IOP, corneal endothelial cell density (ECD) assessed using CEM-530 (Nidek Co., Ltd., Gamagori, Japan) specular microscope, preoperative anterior chamber depth and axial length measured by IOLMaster (Carl Zeiss Meditec AG, Jena, Germany), and intraoperative and postoperative complications. Patients younger than 18 years of age, those who had previously undergone refractive surgery, and those who had been followed for less than 1 year were excluded from the study.

A biconvex polymethylmethacrylate ICIOL (Artisan, Opthec BV, Groningen, Netherlands) 8.5 mm in diameter with a 5.0 mm optical zone was fixed to the anterior iris surface in all patients. IOL power was calculated using IOLMaster (Carl Zeiss Meditec AG, Jena, Germany) with an A-constant of 115.0 and residual myopia of -1.0 D.

### Surgical Technique

Pupillary dilatation was induced in all patients preoperatively by instilling 1 drop of 0.5% tropicamide. All surgical procedures were performed under general anesthesia by the same surgeon (M.K.). Patients who had previously undergone pars plana vitrectomy for any reason and had IOL subluxation or luxation were included in group 1. Group 2 included patients with IOL subluxation only. Patients who had luxated IOL and underwent pars plana vitrectomy during secondary implantation were excluded from the study. In all patients, after opening the conjunctiva, a 23-gauge (G) sclerotomy was made 3.5 mm from the lower temporal limbus and an infusion cannula was placed. Infusion flow was started only when needed. A scleral tunnel 6 mm in diameter was prepared on the 12 o’clock line 2 mm from the limbus, but was not advanced to the anterior chamber. A second 23-G sclerotomy was created 3.5 mm from the limbus in the upper temporal region and the luxated/subluxated IOL was moved into the anterior chamber using forceps. In non-vitrectomized eyes, anterior vitrectomy was performed through this sclerotomy before the IOL was moved into the anterior chamber. In vitrectomized eyes with luxated IOL, illumination was provided transsclerally and a separate sclerotomy was not created. The anterior chamber was accessed via the prepared scleral tunnel and the dislocated IOL was removed. Carbachol 0.01% (Miostat, Alcon, TX, USA) and cohesive viscoelastic substance were administered to the anterior chamber consecutively. Corneal incisions perpendicular to the iris plane were made with a 1-mm blade at the 3 and 9 o’clock positions. The ICIOL was placed in the anterior chamber convex side up. The IOL was stabilized through the scleral tunnel using special forceps (Ophtec Artisan Implantation Standard D02-74 Forceps) and fixated to the iris at 3 and 9 o’clock by aspiration. A peripheral iridectomy was made at 12 o’clock. The scleral tunnel and sclerotomies were sutured with 8/0 vicryl. Four interrupted sutures were used to close the scleral tunnel and one suture was placed at each sclerotomy. After removing the viscoelastic substance, the corneal incisions were made edematous. The conjunctiva was closed with 8/0 vicryl.

Postoperatively, all patients were prescribed topical antibiotic and corticosteroid drops 4 times a day for 1 month. The antibiotic drops were discontinued after 1 month, while the corticosteroid drops were tapered and discontinued within 2 weeks.

### Statistical Analysis

All statistical analyses were performed using the SPSS software package (Version 21, IBM Corp., Armonk, NY, USA). A p value less than 0.05 was considered statistically significant. Mann–Whitney U test was used to compare continuous variables and chi-square test was used to compare categorical data between groups. A Wilcoxon signed-rank test was used for comparisons of preoperative and postoperative 1-year data.

## Results

Indications for previous pars plana vitrectomy in group 1 included rhegmatogenous retinal detachment in 15 eyes (79%), vitreous hemorrhage secondary to proliferative diabetic retinopathy in 1 eye (5.25%), epiretinal membrane in 1 eye (5.25%), macular hole in 1 eye (5.25%), and nucleus dropped into the vitreous cavity during cataract surgery in 1 eye (5.25%).

There was no significant difference between the groups in terms of demographic data, operative time, initial anatomical and functional measurements, or postoperative changes in these measurements (p>0.05 for all) (Table 1).

Preoperative and postoperative data are compared in Table 2 (group 1) and Table 3 (group 2).

There was a significant increase in BCVA in both groups postoperatively (group 1 p=0.01, group 2 p=0.04). Although preoperative BCVA and postoperative letter gain were higher in Group 2 (mean 0.6±0.8 LogMAR, 14.4±26.2 letters) compared to group 1 (mean 0.8±0.6 LogMAR, 9.5±16.3 letters), these differences were not statistically significant (p=0.14, p=0.49). Postoperative SERE was -1.49 diopters in group 1 and -1.32 diopters in group 2. There was no difference between preoperative and postoperative IOP or astigmatism values in either group (p>0.05).

There was no significant difference between the groups in terms of complication rates (p>0.05). None of the patients in either group exhibited rhegmatogenous retinal detachment, epiretinal membrane, cystoid macular edema, choroidal detachment, suprachoroidal hemorrhage, or vitreous hemorrhage perioperatively or postoperatively. ECD was decreased in both groups at postoperative 1 year compared to preoperative measurements (group 1 p<0.001, group 2 p=0.003). There was no difference between the two groups in terms of postoperative decrease in ECD (p=0.7). However, endothelial decompensation occurred in one eye in group 1. This patient had previously undergone a total of six intraocular surgeries, including silicone endotamponade removal procedures due to rhegmatogenous retinal detachment and recurrences. The patient’s anterior chamber depth was 4.02 mm and ECD was 1580 cells/mm^2^ before ICIOL implantation. Hyphema was observed in two eyes in group 1 (10.5%) and in one eye in group 2 (9.1%) on postoperative day 1 (p=0.9) and resolved in all eyes within 1 week without treatment. Corectopia persisting at 1 year was observed in only one eye (5.3%) in group 1 (p=0.4). IOP elevation was detected in the early postoperative period in one eye (5.3%) in group 1 and two eyes (18.2%) in group 2. In one eye in each group, IOP returned to normal levels without medication after discontinuation of the corticosteroid drop used postoperatively, while one eye in group 2 (9.1%) developed secondary glaucoma associated with topical antiglaucomatous drops (p=0.2). IOL dislocation was observed in one eye in both group 1 (5.3%) and group 2 (9.1%) (p=0.7).

## Discussion

Although ICIOL implantation in aphakic eyes is easier and safer than other methods, complication rates vary widely between publications.^6,7,10,11,12,13,14,15,16,17,18,19,20,21,22,23^ These differences may result from variation in surgical histories, placement of the ICIOL in the anterior chamber or retropupillary space, and surgeon experience. Reported complications of ICIOL implantation include endothelial cell loss, corneal decompensation, pigment dispersion, hyphema, transient IOP elevation, secondary glaucoma, IOL dislocation, pupillary block, anterior uveitis, cystoid macular edema, hypotonia, choroidal detachment, retinal detachment, and vitreous hemorrhage.^6,7,10,11,12,13,14,15,16,17,18,19,20,21,22,23^ None of the patients in this study showed pigment dispersion, uveitis, cystoid macular edema, hypotonia, choroidal detachment, retinal detachment, or vitreous hemorrhage within the first postoperative year, while other complications occurred at rates considered acceptable in terms of safety, as stated in the literature.

Corneal decompensation following decreased ECD is one of the most important complications of ICIOLs. The rate of ECD reduction in long-term follow-up after ICIOL implantation has been reported as 6-24%.^10,11,12,14,18,19^ Recently, ICIOLs have mostly been placed in the retropupillary space on the grounds that it leads to less endothelial cell loss. However, studies have revealed no significant difference in ECD decrease between anterior chamber and retropupillary implantation of ICIOLs.^13,16^ Güell et al.^20^ compared eyes that underwent anterior chamber ICIOL implantation with fellow eyes that underwent uncomplicated cataract surgery and observed no difference in ECD at 2 years, although endothelial decompensation occurred in some eyes in the ICIOL group. In eyes undergoing phakic ICIOL implantation, ECD decrease was found to be greater in eyes with anterior chamber depth of <3.0 mm compared with those with anterior chamber depth of >3.40 mm,^24^ but there is no study demonstrating the same phenomenon in aphakic eyes. In the present study, corneal decompensation was observed in one eye (3.3%) with an anterior chamber depth of 4 mm and preoperative ECD of 1580 cells/mm^2^. We speculated that the corneal decompensation may have been due to the total of six vitreoretinal surgeries this eye had undergone before ICIOL implantation.

Because it is more difficult to maintain a stable IOP during surgery in vitrectomized eyes, secondary implantation surgeries are more susceptible to complications. The present study showed that complication rates did not differ between the vitrectomized and non-vitrectomized eyes of patients who underwent concurrent dislocated IOL extraction and ICIOL implantation. Labeille et al.^13^ observed a 20.5% mean ECD reduction in the first 3 months in eyes that underwent concurrent ICIOL implantation and pars plana vitrectomy due to a dislocated nucleus or IOL. They reported that using an endofragmatome did not cause greater endothelial loss. However, their operative time was calculated as 72 minutes if the surgery was performed within 2 days of dislocation and 60 minutes if performed after 2 days, which is much longer than the mean operative time of 26.4 minutes in the present study. They also reported complications that were not observed in our study, such as cystoid macular edema (25%), retinal detachment (12.5%), vitreous hemorrhage (12.5%), and choroidal detachment (3%), at higher rates than other studies that employed a similar surgical procedure.^6,23^ This difference may be related to operative time. In two studies conducted in vitrectomized aphakic eyes instead of eyes with dislocated IOLs as in our study, Acar et al.^18^ reported a 24% decrease in ECD over a mean follow-up period of 15 months, while Riazi et al.^19^ reported an ECD decrease of 8.1% at 6 months. When all of the eyes in our study were taken into account, the decrease in ECD at 1 year after simultaneous dislocated IOL removal and ICIOL implantation was 12.9%, consistent with the literature. Furthermore, the vitrectomized and non-vitrectomized eyes in our study showed no significant difference in ECD decrease.

In a study including 148 vitrectomized eyes, epiretinal membrane, proliferative vitreoretinopathy, pupillary capture of the IOL, endophthalmitis, and choroidal hemorrhage were reported after secondary scleral fixation IOL implantation in addition to the ICIOL-related complications described in the literature.^25^ A comparison of ICIOL implantation and scleral fixation IOL implantation performed concurrently with pars plana vitrectomy showed that ICIOLs yielded better corrected and uncorrected visual acuity.^3^

In our study, BCVA increased postoperatively in both groups. Studies comparing anterior chamber and retropupillary ICIOLs revealed no differences in BCVA.^15,16^ Postoperative astigmatism was found to be lower in patients who underwent scleral tunnel incision compared to those who had corneal incisions. Accordingly, uncorrected visual acuity was higher in the scleral tunnel incision group.^16^ In the present study, we achieved both low postoperative astigmatism by using scleral tunnel incision and good IOP stability by not opening the connection between the tunnel and anterior chamber until moving the dislocated IOL into the anterior chamber. We also showed that, as with other parameters, there was no difference between vitrectomized and non-vitrectomized eyes in terms of change in astigmatism.

The complete absence of complications such as cystoid macular edema, hypotonia, choroidal detachment, retinal detachment, and vitreous hemorrhage in our study may be attributed to minimizing operative time by making as few manipulations as possible and ensuring good stabilization of the anterior chamber.

Increased IOP after ICIOL implantation may occur due to the use of corticosteroid drops, inadequate iridectomy, pigment dispersion, or surgical trauma, and has been reported at rates of 2.6-11.4% in the literature.^6,16,17,22^ The prevalence of secondary glaucoma is 0-6.2%.^6,16,17,22,23^ In the present study, IOP elevation was observed in three patients (10%) in the early postoperative period. IOP returned to normal levels in two of these patients without medication after discontinuing corticosteroid drops, but one patient (3.3%) developed secondary glaucoma. There was no difference between vitrectomized and non-vitrectomized eyes in terms of IOP elevation and glaucoma development.

### Study Limitations

The limited number of patients, retrospective design, and lack of a retropupillary ICIOL group are limitations of this study. More comprehensive prospective studies may provide insight into issues that remain uncertain.

## Conclusion

As in non-vitrectomized eyes, simultaneous dislocated IOL extraction and secondary ICIOL implantation in the anterior chamber is a fast and safe surgical procedure in vitrectomized eyes as well. In these patients, aspiration can be used for iris enclavation. Excellent postoperative astigmatism results can be obtained with scleral incision.

## Figures and Tables

**Table 1:**
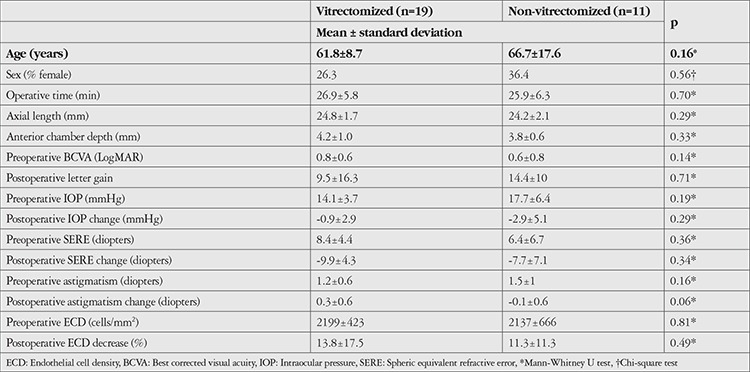
Between-group comparisons

**Table 2:**
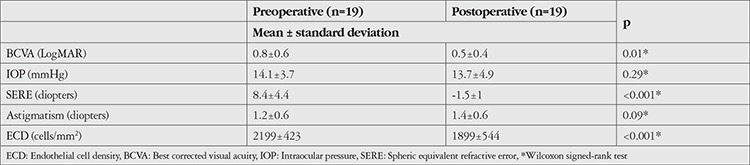
Comparison of preoperative and postoperative data in vitrectomized eyes

**Table 3:**
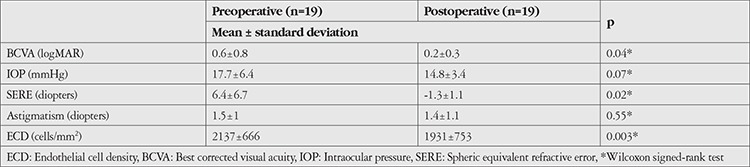
Comparison of preoperative and postoperative data in non-vitrectomized eyes

**Figure 1:**
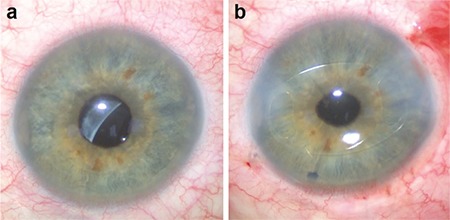
Images of a 58-year-old vitrectomized patient with intraocular lens (IOL) dislocation. a) preoperative image shows that the IOL is dislocated together with the capsular bag; b) postoperative image shows an iris-claw IOL placed in the anterior chamber and peripheral iridectomy
